# Surgery for pancreatic neuroendocrine tumors during the COVID-19 pandemic: a retrospective cohort from a high-volume center

**DOI:** 10.1007/s13304-024-01942-z

**Published:** 2024-07-21

**Authors:** Salvatore Paiella, Luca Landoni, Matteo De Pastena, Giovanni Elio, Fabio Casciani, Sara Cingarlini, Mirko D’Onofrio, Giulia Maistri, Ivan Ciatti, Massimiliano Tuveri, Maria Vittoria Davì, Claudio Luchini, Katia Donadello, Gessica Manzini, Giuseppe Malleo, Roberto Salvia

**Affiliations:** 1 Pancreatic Surgery Unit, Pancreas Institute, University of Verona, Policlinico GB Rossi, Piazzale Scuro 10, 37134 Verona, Italy; 2 Oncology Unit, Pancreas Institute, University of Verona, Verona, Italy; 3 Radiology Unit, Pancreas Institute, University of Verona, Verona, Italy; 4 Department of Medicine, Section of Endocrinology, University and Hospital Trust of Verona, Verona, Italy; 5 Department of Diagnostics and Public Health, Section of Pathology, University of Verona, Verona, Italy; 6 ARC-Net Research Center for Applied Research on Cancer, University of Verona, Verona, Italy; 7 Anaesthesia and Intensive Care Unit B, University of Verona Hospital Trust, Verona, Italy; 8 Surgical Block, University of Verona Hospital Trust, Verona, Italy

**Keywords:** Pancreatic neuroendocrine tumors, COVID-19, SARS-CoV2, Pancreatic surgery, Pancreatic tumors

## Abstract

During the COVID-19 pandemic, pancreatic surgery for pancreatic neuroendocrine tumors (PNETs) with surgical indications was postponed or canceled. Patients with PNET patients who underwent pancreatic surgery during the COVID-19 restriction period (3 years) were compared with a similar cohort of patients who underwent surgery in the previous 3 years. Data on patients’ characteristics, waiting time, and surgical and pathology outcomes were evaluated. During the study period, 370 patients received surgery for PNETs, 205 (55%) during the first period, and 165 (45%) during the pandemic. A lengthening of the waiting list (182 [IQR 100–357] vs. 60 [40–88] days, *p* < 0.001) and increased use of anti-tumor medical treatments (any therapy, peptide receptor radionuclide therapy, and somatostatin analogs; all *p* < 0.001) was found. During the pandemic, surgery occurred after a median of 381 days [IQR 200–610] from diagnosis (vs. 103 [IQR 52–192] of the pre-COVID-19 period, *p* < 0.001). No statistically significant differences in tumor size and grading distribution were found between the two periods (both *p* > 0.05), yet only a modest increase of the median Ki67 values in cases operated during the pandemic (4% vs. 3%, *p* = 0.03). Lastly, these latter patients experienced less major postoperative complications (13% vs. 24%, *p* = 0.007). During COVID-19, the surgical waiting list of PNET patients was drastically extended, and bridge therapies were preferred. This did not result in more advanced cases at final pathology. PRRT and SSA are valid alternative therapies for PNETs when surgery is not feasible.

## Introduction

The COVID-19 pandemic had a significant impact on the healthcare system in Italy, also modifying the management of cancer patients. The pandemic caused a surge in demand for hospital beds and resources, which strained hospitals’ ability to provide adequate care to all patients, including those with cancer [1].

Disruptions occurred in oncological surgical activities as well. The reallocation of resources to manage the surge in COVID-19 patients led to the prioritization of urgent and critical cancer surgeries, while elective surgeries were often postponed or canceled. This resulted in missed surgeries and long waiting lists, which can have significant consequences for cancer patients, potentially delaying treatment, increasing the risk of cancer progression, and causing anxiety and distress for many cancer patients [2–4]. In parallel, several bioethical concerns were raised. One of the most controversial issues is selecting patients for treatment when resources are scarce [4, 5]. Some have argued that it is ethical to prioritize young patients with no comorbidities and a more favorable prognosis [6]. However, others have argued that this approach would violate the principles of equity and solidarity, as it would unfairly disadvantage older patients and those with comorbidities [7].

The complexity of pancreatic surgery requires careful selection of the patients most likely to benefit from the operation. The decision of whether or not to operate on a patient with a pancreatic tumor depends on several factors, including the stage of the cancer, the patient’s overall health, and the availability of resources (e.g., intensive care unit).

The The Pancreatic Surgery Unit of the Pancreas Institute of the University of Verona Hospital Trust is a third-level, very high-volume centre for pancreatic surgery. During the pandemic, surgical activity has been drastically rearranged [6], prioritizing malignant conditions already treated with neoadjuvant therapy. This excluded other borderline-malignant conditions requiring surgery, such as non-functioning pancreatic neuroendocrine tumors (NF-PNETs) [7].

NF-PNETs are a heterogeneous group of rare, typically indolent tumors that, when > 20 mm, require surgery to improve prognosis and prevent recurrence [8]. During the pandemic, the management of NF-PNET was heavily impacted, with delayed diagnoses, disrupted surveillance, postponed surgeries and nonsurgical therapies [9, 10].

This study aimed to depict how the COVID-19 pandemic impacted the management of NF-PNETs in a referral, very high-volume center of pancreatic surgery.

## Methods

### Study design

A retrospective analysis was conducted in consecutive patients who underwent pancreatic surgery with a final diagnosis of NF-PNET. Patient data were retrieved from the institutional prospectively maintained database. The COVID-19 period was compared to a similarly long period immediately preceding the pandemic. The institutional management of PNETs has been reported elsewhere [8, 11, 12].

### Data collection

Electronic and paper medical records were reviewed to collect the following data: demographic (age, sex, BMI, ASA score), NF-PNET characteristics (tumor dimensions at diagnosis and preoperatively, tumor site and stage, any previous oncological treatments [peptide radionuclide therapy—PRRT, somatostatin analogs—SSA, chemotherapy, radiotherapy], surgical (days of waiting from diagnosis to surgery, type of surgery), postoperative (any major postoperative complications—Clavien-Dindo ≥ 3[13]), and final pathology (tumor dimensions, tumor grading and Ki67, nodal status, R-status, and lymph-vascular invasion). Waiting time was defined as the time from the indication to surgery at the multidisciplinary tumor board to surgery. Institutional management of PNETs has been previously reported elsewhere [8]. Briefly, PNETs undergo cross-sectional imaging (preferably with magnetic resonance), endoscopic or percutaneous biopsy fine-needle aspiration or biopsy for diagnosis confirmation and Ki67 assessment, ^68^Gallium DOTA-PET/CT (^18^FDG PET/TC is selectively prescribed), and multidisciplinary evaluation.

### Statistics

Continuous variables were expressed as medians with interquartile range (IQR). The groups were compared for categorical variables using the Chi-square or Fisher’s exact tests. For non-normally distributed continuous variables, the Mann–Whitney *U* test was employed. Statistical significance was defined as *p* ≤ 0.05. Data were analyzed using SPSS software release 25 (SPSS and IBM company, Armonk, NY).

## Results

### Baseline characteristics

The study population consisted of 370 patients. Among these patients, 205 (55%) underwent surgery between March 2017 and March 2020 (the period before the lockdown in Italy), while 165 underwent surgery during the COVID-19 era (March 2020 to March 2023). There were no clinically significant differences in baseline characteristics between the two periods. However, during the COVID-19 era, a higher frequency of preoperative oncological treatments, known as “bridge therapy,” was observed (any therapy, PRRT, SSA, and chemotherapy; all *p* < 0.001). Table 1 presents the baseline characteristics.

**Table 1:** Baseline characteristics

	Pre-COVID-19, 205 (55%)	During COVID-19, 165 (45%)	*p* value
Age (years, IQR)	56 [47–65]	54 [48–66]	0.309
Sex (female), *n* (%)	103 (50)	66 (40)	**0.031**
BMI, kg/m^2^ (median, IQR)	26 [23–28]	25 [23–28]	0.979
ASA score > II, *n* (%)	26 (13)	22 (13)	0.487
Tumor location, *n* (%)			0.380
Head	84 (41)	71 (43)	
Body tail	121 (59)	94 (57)	
Bridge therapy (any), *n* (%)*	43 (21)	76 (46)	** < 0.001**
PRRT	18 (9)	40 (24)	** < 0.001**
SSA	27 (13)	53 (32)	** < 0.001**
Imaging features at diagnosis			
Tumor size, mm (median, IQR)	23 [14–36]	30 [16–40]	0.248
Vascular infiltration at imaging, *n* (%)	29 (14)	23 (14)	0.539
Suspected N1 at imaging, *n* (%)	31 (15)	39 (24)	**0.024**
Imaging features at preoperative radiological reassessment			
Tumor size, mm (median, IQR)	25 [16–35]	28 [19–39]	0.091
Vascular infiltration at imaging, *n* (%)	13 (6)	25 (15)	**0.005**
Suspected *N*1 at imaging	31 (15)	32 (19)	0.177

Values in bold are statistically significant

*BMI* body mass index, *ASA* American Society for Anesthesiologist, *PRRT* peptide receptor radionuclide therapy, *SSA* somatostatin analogs, *IQR* interquartile range

*Patients might have received multiple therapies

Among the radiological parameters considered, a higher prevalence of suspected vascular infiltration (including simple contact with a major peripancreatic vessel, 15% vs. 6%, *p* = 0.005) and metastatic lymphadenopathies (24% vs. 15%, *p* = 0.024) was observed at diagnosis during the COVID-19 period.

### Surgical and postoperative data

Table 2 presents surgical and postoperative details. The median time from diagnosis to surgery increased from 182 (IQR 100–357) to 382 days (IQR 200–610), *p* < 0.001, and the waiting list duration increased from 60 (IQR 40–88) to 103 days (52–192), *p* < 0.001. No differences were found in the surgical variables considered, apart from the operative time slightly increasing during the second period, from a median of 383 (IQR 284–468) to 400 (IQR 320–470) minutes. Major postoperative complications were less frequent during COVID-19 (24% vs. 13%, *p* = 0.007).

**Table 2:** Surgical and postoperative data

	Pre-COVID-19, 205 (55%)	During COVID-19, 165 (45%)	*p* value
Time from diagnosis to surgery, days, median (IQR)	182 [100–357]	382 [200–610]	** < 0.001**
Waiting list duration, days (median, IQR)	60 [40–88]	103 [52–192]	** < 0.001**
Type of resection, *n* (%)			0.148
Pancreaticoduodenectomy	69 (33.7)	57 (34.5)	
Left pancreatectomy	6 (2.9)	10 (6.1)	
Total pancreatectomy	101 (49.3)	85 (51.5)	
Other surgeries	29 (14.1)	13 (7.9)	
Surgical approach, *n* (%)			0.348
Open	138 (67)	107 (65)	
Minimally invasive	67 (33)	58 (35)	
Vascular resection (any), *n* (%)	7 (3)	9 (6)	0.241
Multivisceral resection, *n* (%)	34 (17)	32 (19)	0.285
Major postoperative complications, *n* (%)	49 (24)	22 (13)	**0.007**

Values in bold are statistically significant

*IQR* interquartile range

### Pathology data

Data are presented in Table 3*.* During the COVID-19 period, no statistically significant differences were found in the grading of the tumors. The median Ki67 was 4 (IQR 2–8) during the COVID-19 era and 3 (IQR 2–6) during the previous period (*p* = 0.03). During the pandemic, final pathology revealed lower and higher rates of microvascular and lymphatic infiltration, respectively (both *p* < 0.05).

**Table 3:** Pathology data

	Pre-COVID-19, 205 (55%)	During COVID-19, 165 (45%)	*p* value
Tumor size, mm (median, IQR)	24 [15–35]	28 [18–40]	0.095
Microvascular infiltration, *n* (%)	67 (34)	88 (56)	< 0.001
Perineural infiltration, *n* (%)	63 (32)	55 (35)	0.288
Lymphatic infiltration, *n* (%)	56 (28)	26 (17)	0.008
Harvested lymph nodes (median, IQR)	26 [11–36]	26 [18–36]	0.879
N1, *n* (%)	86 (42)	80 (42.4)	0.349
R1, *n* (%)	16 (8)	18 (11)	0.306
Grading*			0.172
G1, *n* (%)	106 (55.5)	69 (44.2)	
G2, *n* (%)	75 (39.3)	77 (49.4)	
G3, *n* (%)	10 (5.2)	10 (6.4)	
Ki67 (median, IQR)*	3 [2–6]	4 [2–8]	**0.030**
TNM staging			0.689
Stage I, *n* (%)	40 (19.5)	27 (16.5)	
Stage II, *n* (%)	62 (30.2)	49 (29.9)	
Stage III, *n* (%)	103 (50.2)	89 (54.3)	

Values in bold are statistically significant

*IQR* interquartile range

*Data available for 191 (93.2%, pre-COVID-19 group) and 156 (94.5%, during COVID-19 group) patients

## Discussion

COVID-19 profoundly transformed health infrastructures, necessitating adaptations to meet the surge in healthcare demands, optimize resource allocation, and prioritize the needs of oncological patients. Pancreatic surgery units focused on malignant conditions, relegating benign or low/intermediate risk tumors to low priority [14]. PNETs exemplify this paradigm shift. During the pandemic, the North American Neuroendocrine Tumor Society Guide for Neuroendocrine Tumors (NANETs) promptly recognized the significant impact of the pandemic on the management of PNET, providing guidelines for managing these tumors [15]. Patients with resectable PNETs with an indication for surgery were recommended to start SSA and other anti-tumor medical treatments as an alternative, postponing surgery according to the severity of the impact of COVID-19 in the hospital [15].

We report a statistically significant lengthening of the waiting list, and PNET patients received surgery after a median of 182 days (IQR 100–357) vs. 60 (IQR 40–88) of the pre-COVID period (*p* < 0.001). This has already been reported by other authors, where surgeries for low-grade PNETs and other non-malignant conditions were postponed in favor of malignant ones [16].

The results of this study confirm previous findings on the management of neuroendocrine tumors in Italy during the pandemic, where patients were addressed mostly to anti-tumor medical treatments [9, 10]. In the surgical cohort examined here, the use of a bridge therapy more than doubled (46% vs. 21%), especially for SSA (32% vs. 13%, both *p* < 0.001), compared to the pre-COVID-19 era. This is in line with the relevant increase in the time between diagnosis and surgery (163 days [IQR 52–192] vs 382 [IQR 200–610], *p* < 0.001). Nonconventional bridge therapy can be a valid ally of pancreatic surgery when surgery is not amenable or during the waiting list, for PNETs that have shown significant changes (e.g., an abrupt increase in dimensions during follow-up or symptoms appearance) or that already have a concerning biology (e.g., G2 tumors with high levels of Ki67).

Favoring nonsurgical treatments did not seem to impact tumor biology negatively. Indeed, at final pathology, only a statistically significant, yet non-clinically relevant, higher Ki67 was found (4% vs. 3%, *p* = 0.03). Time will be necessary to assess if this, and other parameters, will impact negatively prognosis.

Notably, the rate of major postoperative complications decreased from 24 to 13%. We do not have a clear explanation for this. It might be due to the positive effects of PRRT on the development of pancreatic fistulas, as already postulated [17], and similarly to what has been reported in patients with pancreatic cancer treated with neoadjuvant therapy [18]. Another explanation may be that during the pandemic, for pancreatic head tumors the long waiting list favored pancreatic duct’s dilation and higher pancreatic fibrosis, both protective factors for pancreatic fistula. However, these are speculations deserving further investigations.

To conclude, in moments of resource constraints, such as during the COVID-19 pandemic, anti-tumor medical treatments may be a temporary valid therapy for well-differentiated low-grade PNETs. In particular, PRRT and SSA seem to freeze PNETs, avoiding significant tumor progression. Any long-term effects of this PNETs management, detrimental or positive, will need to be proven appropriately.

## Data Availability

All data generated or analyzed during this study are included in this article. Further enquiries can be directed to the corresponding author.
